# Universal, school-based transdiagnostic interventions to promote mental health and emotional wellbeing: a systematic review

**DOI:** 10.1186/s13034-024-00735-x

**Published:** 2024-04-10

**Authors:** Peng Wang, Zhaoqi Wang, Shuiwei Qiu

**Affiliations:** 1 Moray House School of Education and Sport, University of Edinburgh, Edinburgh, UK; 2 School of Foreign Studies, China University of Petroleum, Qingdao City, China; 3 Department of Cardiothoracic Surgery, Quzhou People’s Hospital, Quzhou City, China; 4 Department of Language, Literature and Communication, Faculty of Humanities, Vrije Universiteit Amsterdam, Amsterdam, Netherlands; 5 Department of Psychology, Education and Child Studies, Erasmus School of Social and Behavioural Sciences, Erasmus University Rotterdam, Rotterdam, Netherlands

**Keywords:** Transdiagnostic, Intervention, Promotion, Mental health, Emotional wellbeing

## Abstract

**Objective:**

This systematic review aims to evaluate the effectiveness of universal school-based transdiagnostic interventions in promoting the mental health of children and adolescents. It compares and discusses interventions targeting the prevention of mental disorders versus the promotion of mental health. Additionally, the roles of teachers and psychologists as intervention conductors are examined.

**Methods:**

A comprehensive search of the Psycinfo, Pubmed, and Web of Science databases was conducted without any time restrictions to identify relevant literature on universal school-based transdiagnostic interventions promoting children and adolescents' mental health.

**Results and discussion:**

The findings reveal that universal school-based transdiagnostic promotion/prevention programs have a small to medium overall effect size. These interventions demonstrate a broad coverage of different aspects of children and adolescents' mental health. However, the relative effectiveness of teacher-led versus psychologist-led interventions remains unclear. Interventions focused on preventing mental disorders exhibit a higher effect size, albeit on a narrower range of mental health aspects for children and adolescents.

**Significance:**

This study enhances our understanding of universal school-based transdiagnostic interventions and their impact on children and adolescents' mental health. Further research is needed to elucidate the comparative efficacy of teacher-led and psychologist-led interventions and to explore the specific dimensions of mental health targeted by these interventions.

## Introduction

The mental health of children and adolescents is crucial for their overall development. Poor mental health can have varying impacts on their daily lives, academic performance, and prospects, depending on its severity [1]. Severe cases may even lead to suicidal behaviors [2]. It is important to note that the effects of mental ill-health during the early years can persist into adulthood and have a lasting influence on individuals' lives [3]. With the significant prevalence and persistence of mental disorders, it is alarming that approximately one-fifth of children and adolescents worldwide have experienced one or more clinical disorders, and more than half have shown sub-clinical symptoms [4].

Given that children and adolescents typically spend a significant amount of their daily time in educational settings, school-based mental health services have emerged as a critical battleground for addressing their mental well-being [5]. However, the current provision of school-based mental health services falls short of meeting the needs of children and adolescents, particularly in low- and middle-income countries (LMICs). Limited access to services and the compatibility of available mental health services with the specific needs of young individuals contribute to this insufficiency and inefficiency [6].

In recent decades, there has been significant progress in developing mental health promotion programs for children and adolescents [7]. These programs primarily focus on school-based universal interventions, demonstrating positive effects on the mental health of young individuals [8]. However, systematic reviews and meta-analyses examining the efficacy of these interventions have shown mixed results [9]. Moreover, prevailing single-diagnosis protocols limit the effectiveness of these programs in addressing co-occurring disorders that commonly exist among individuals [10].

Despite the potential benefits of universal school-based interventions, there is an ongoing debate regarding their effectiveness compared to targeted interventions. Targeted interventions are designed for individuals or groups identified as at-risk for developing mental health issues, whereas universal interventions are applied to all students regardless of their risk level. Literature suggests that targeted interventions may offer more significant benefits for those at high risk, potentially due to their focus on specific needs and challenges [11–13]. However, universal interventions play a crucial role in promoting mental health and well-being across the entire student population, aiming to prevent the onset of mental health issues before they arise. This approach is especially relevant in educational settings, where identifying and targeting at-risk individuals can be challenging. Thus, examining the effectiveness of universal interventions remains worthwhile, particularly in their potential to create inclusive, supportive environments that benefit all students.

To overcome these challenges and enhance the efficiency of mental health promotion, a growing emphasis has been placed on adopting a transdiagnostic framework for children and adolescents. Transdiagnostic interventions offer a promising approach that goes beyond targeting specific disorders and focuses on promoting overall mental health and emotional well-being [14]. By addressing a broader range of factors related to mental health, such as mental health literacy, emotions, behaviors, social management techniques, values, and perceptions, transdiagnostic interventions aim to meet the diverse needs of most children and adolescents [15, 16]. Specifically, 'values' here refer to the core beliefs and principles that influence children and adolescents' attitudes and behaviors towards mental health, encompassing what they deem important in their lives and schooling experience. 'Perceptions,' meanwhile, encompass their understanding and attitudes towards mental health issues, including awareness, stigma, and openness to intervention.

While previous reviews have highlighted the positive influence of school-based universal interventions on important determinants of children and adolescents' mental health, adopting transdiagnostic protocols can further enhance the overall effectiveness [8]. Transdiagnostic interventions have the potential to provide significant benefits by targeting a wider range of mental aspects and addressing co-occurring disorders in a more comprehensive manner. Therefore, there is an urgent and compelling need to conduct a systematic review of existing universal school-based transdiagnostic interventions to evaluate their efficacy in promoting the mental health and well-being of children and adolescents.

This article seeks to contribute to the existing body of knowledge by conducting a thorough systematic review of transdiagnostic interventions. By carefully analyzing the available evidence, our aim is to highlight the potential benefits associated with transdiagnostic interventions and shed light on their ability to address the limitations inherent in conventional single-diagnosis protocols. The insights gained from this comprehensive review will provide policymakers, educators, and mental health professionals with valuable information to inform their decision-making processes and facilitate the development of more effective strategies that promote the mental well-being of children and adolescents in a holistic manner.

### Definitions of transdiagnostic constructs

Transdiagnostic treatments are a range of therapies that can be applied broadly across different disorders [17]. The foundation of transdiagnostic treatments lies in transdiagnostic constructs, which are the underlying mechanisms that cut across various disorders (Stanton et al. 2020). In this context, there are two types of transdiagnostic constructs: descriptively transdiagnostic processes and mechanistically transdiagnostic processes.

Descriptively transdiagnostic processes are observed across multiple disorders, indicating their presence in a range of conditions [18]. For instance, the study by Mehrdadfar et al. [19] revealed that an online transdiagnostic treatment effectively enhanced social-emotional skills, such as emotional competence, in children with cochlear implants, illustrating emotional competence's broad relevance across various conditions.

On the other hand, mechanistically transdiagnostic processes are the ones that provide causal and functional explanations for the co-occurrence of disorders [20]. An example of a mechanistically transdiagnostic process is the online Brief Emotion Regulation Training (BERT) program for emerging adults developed by Gatto et al. [21]. The program, rooted in cognitive-behavioral practices, aimed to improve emotional competence, demonstrating its transdiagnostic significance by showing that improved emotion regulation can causally influence mental wellness.

To further illustrate the distinction between these concepts, two common transdiagnostic constructs are exemplified: self-esteem and rumination [22]. Self-esteem has been found to be present in various mental disorders such as social anxiety disorder and schizophrenia, indicating its descriptively transdiagnostic nature [23, 24]. However, whether self-esteem is mechanistically transdiagnostic remains uncertain without evidence supporting its causal impact on mental disorders. In contrast, rumination is believed to have a detrimental effect on emotional well-being and can causally influence it, leading to a vicious cycle of worsening behavior [25, 26], namely, normally believed to be mechanistically transdiagnostic.

### Transdiagnostic interventions

Based on the identification of transdiagnostic constructs, transdiagnostic interventions have been developed in recent years [22]. These interventions can be broadly categorized into two types based on their developmental basis. The first type involves targeting the identified transdiagnostic constructs to improve treatment efficacy from a psychopathology perspective [27]. The second type involves extending existing single-diagnosis treatments by adapting their protocols to be applicable across disorders, with cognitive behavioral therapy (CBT) being a primary example [28]. While many universal school-based interventions aimed at promoting children and adolescents' mental health by nurturing their social-emotional learning (SEL) abilities fall into the first type [29], they are often not explicitly identified as transdiagnostic in previous review articles [30, 31].

The efficacy of transdiagnostic interventions developed from single-diagnosis treatments is intertwined with their effectiveness in treating psychopathology. Since these interventions build upon the foundation of existing single-diagnosis protocols, psychologists may already possess a significant portion of the necessary knowledge [32]. In contrast, teachers, who are often the conductors of school-based interventions, need training to acquire the relevant intervention knowledge, and it is unclear whether this new acquisition would affect intervention efficiency. Considering the complexity of transdiagnostic theory, it is essential to examine whether teacher-led or psychologist-led interventions are more effective.

However, the binary classification of transdiagnostic interventions mentioned above does not adequately explain the advantages and disadvantages of different interventions in a methodological manner. Therefore, this chapter will adopt the classification proposed by Sauer-Zavala et al. [22] to present the current transdiagnostic approaches and existing debates. Under this framework, transdiagnostic interventions are divided into three types: those with universally applied therapeutic principles, modular treatments, and shared mechanism treatments. Modular treatments, observed at an individual level, will not be discussed in the present study.

#### Universally applied therapeutic principles

Interventions that utilize universally applied therapeutic principles are grounded in specific theoretical frameworks, such as cognitive-behavioral treatment [33]. These interventions are characterized by their ability to be applied across a variety of psychopathologies using a single protocol, showcasing their universal applicability [34]. While these types of interventions have long been used in the treatment of mental disorders, their classification as transdiagnostic is a more recent development [35]. This shift in understanding has been driven by an increased awareness of their impact on co-occurring disorders. Many widely recognized transdiagnostic treatments fall into this category, as they incorporate existing treatments and minimize the need for the creation of new interventions [10].

Interventions based on universally applied therapeutic principles are structured to broadly address psychological disorders, utilizing standardized protocols that are effective across a diverse range of conditions. This differs from grouping them based on shared mechanisms or psychological processes. This "top-down" approach, referred to as a "one size fits all" strategy by Sauer-Zavala et al. [22], contrasts with the "bottom-up" approach of shared mechanism treatments, which target shared mechanisms across disorders and formulate treatment protocols based on these mechanisms.

A potential limitation of interventions with universally applied therapeutic principles is that their impact on co-occurring disorders is not always intentional. Many of these interventions were originally designed to address specific mental disorders, and their effects on other co-existing disorders may be incidental rather than by design. However, with the rise of transdiagnostic awareness, some of these interventions have been adapted to more effectively address co-occurring disorders, compensating for their initial lack of specificity. Despite this limitation, interventions with universally applied therapeutic principles offer several advantages. Firstly, as they are often based on existing protocols, their development is less time-consuming compared to creating entirely new approaches. Secondly, it is easier for practitioners, such as teachers or psychologists, to acquire the necessary knowledge, which is especially beneficial for school-based interventions. As a result, interventions with universally applied therapeutic principles are of significant interest and are expected to be widely utilized in universal school-based practice.

##### Mindfulness-based interventions

Mindfulness-based interventions aim to cultivate a state of peaceful awareness and encourage individuals to focus on the present moment of internal and external experiences (Greenberg & Harris, 2012). These interventions typically involve body-focused meditation or movement practices where recipients are instructed to focus on their breathing or current movements [36]. Mindfulness is considered a form of emotion regulation and has been widely disseminated in the adult population to promote mental health and emotional well-being, particularly in non-clinical settings [37]. Recent studies have shown that mindfulness-based interventions can be effective as transdiagnostic treatments, addressing a variety of mental health problems by targeting underlying mechanisms such as rumination and worry [38].

Mindfulness-based interventions have advantages in terms of simplicity and group-friendliness. The instructions are relatively straightforward, reducing the requirements for conducting the intervention. Additionally, the instructions can be applied to a broad population, making these interventions suitable for group settings. In the context of school-based interventions, mindfulness-based approaches do not require a lengthy adjustment period, can be easily adopted by individuals, and do not pose risks of worsening mental health. Furthermore, teachers or psychologists can acquire the necessary knowledge to deliver these interventions with relative ease. Therefore, mindfulness-based interventions have a clear advantage in universal school-based practice and are expected to be widely employed.

##### Acceptance and commitment therapy (ACT)

Acceptance and Commitment Therapy (ACT) is a comprehensive modality that focuses on experiential avoidance as a central difficulty associated with emotional distress, such as anxiety, depression, and stress [39]. ACT aims to enhance individuals' psychological flexibility, which involves their ability to persist in or maintain their current mental states in response to challenging environments [40]. ACT employs various core techniques that collectively work to develop psychological flexibility. Recent research has shown that ACT can be used as a transdiagnostic intervention, addressing a broad range of mental states and disorders by targeting underlying mechanisms such as psychological inflexibility [41].

The core techniques of ACT include:

1. Encouraging individuals to maintain balance and broaden their scope of reaction to negative thoughts and feelings, thereby protecting them from maladaptive avoidance behaviors.
2. Using psychoeducation to strengthen individuals' commitment to positive values in their lives, such as family, work, and friendships.
3. Emphasizing commitment and consistency in actions aligned with personal values.
4. Promoting willingness to accept adverse events and feelings by utilizing emotional resources developed through previous skills.
5. Fostering workability through psychological flexibility.

ACT, as a newly developed transdiagnostic intervention, addresses a broader range of mental states than many other transdiagnostic approaches, making it promising for meeting heterogeneous needs across single-diagnosis and general mental health contexts [42]. It has been associated with improvements in mental health and overall well-being [43]. Furthermore, ACT is structured in sessions, making it suitable for integration into school curricula. Therefore, ACT is expected to be frequently encountered in the current systematic review, as it offers a comprehensive approach to address various mental health concerns.

##### Social emotional learning (SEL) interventions

Social Emotional Learning (SEL) interventions are another type of universally applied therapeutic principles that aim to foster the development of five core competencies: self-awareness, self-management, social awareness, relationship skills, and responsible decision-making [44]. These competencies are believed to be critical for mental health, well-being, and success in life [29].

SEL interventions are designed to be universally applicable and can be implemented in various settings, including schools, to promote the emotional and social competence of children and adolescents [29]. They are based on the understanding that learning is a social and emotional process, and that these skills can be taught in a coordinated manner [45].

A study by Mehrdadfar et al. [19] demonstrated the transdiagnostic nature of SEL interventions. The study found that a unified protocol for online transdiagnostic treatment improved social-emotional skills, including emotional competence, in children with cochlear implants. This suggests that SEL interventions can be effective across a range of conditions and disorders, highlighting their descriptively transdiagnostic nature.

Furthermore, Gatto et al. [21] developed an online Brief Emotion Regulation Training (BERT) program for emerging adults, rooted in cognitive-behavioral practices, which aimed to improve emotional competence. The program demonstrated its transdiagnostic significance by showing that improved emotion regulation can causally influence mental wellness, indicating the mechanistically transdiagnostic nature of SEL interventions.

SEL interventions, like Mindfulness-based interventions and Acceptance and Commitment Therapy (ACT), have the potential to address a broad range of mental states and meet heterogeneous needs across single-diagnosis and general mental health contexts. Therefore, they are expected to be frequently encountered in the current systematic review, as they offer a comprehensive approach to address various mental health concerns.

#### Shared mechanisms treatment

Shared mechanisms treatment represents a transformative shift in the field of transdiagnostic interventions, aiming to identify and target shared constructs and processes that underlie multiple disorders [22]. It explicitly focuses on identifying and influencing the common mechanisms that cut across different disorders, distinguishing itself from other types of interventions. This approach builds upon the conceptualization of mechanistically transdiagnostic constructs and informs treatment protocols by identifying core processes [22].

Three methods typically represent existing programs of shared mechanisms treatment:

1. Exposure practice: This approach primarily targets anxiety disorders and involves gradually or intensely exposing individuals to feared stimuli [46]. By addressing the overestimation of potential risks and dangers associated with these stimuli, exposure practice aims to facilitate fear habituation, disconfirmation of fear beliefs, and promote inhibitory learning [32]. While exposure practice may not be suitable for school-based settings due to its environmental requirements and time-consuming nature, it remains a valuable intervention.
2. Cognitive-Behavioral Therapy-Enhanced (CBT-E): Developed as a shared-mechanism treatment for eating disorders, CBT-E focuses on changing individuals' awareness regarding the overvaluation of weight and shape [47]. This approach involves reducing weight checking frequency and improving diet plans. However, CBT-E requires multifaceted assistance from various sources, making it less applicable in pure school-based delivery modes.
3. Unified Protocol (UP) for Transdiagnostic Treatment of Emotional Disorders: UP targets shared mechanisms across emotional disorders and explicitly aims to address their co-occurrence. For example, UP focuses on reducing reactive distress triggered by strong negative emotions and preventing reliance on avoidant coping strategies. It incorporates elements of mindfulness-based interventions, psychological flexibility, and raising relevant awareness [48]. While UP is comprehensive, its implementation requires access to relevant training and resources for both conductors and recipients, making it less feasible for widespread school-based use.

Considering the accessibility, training requirements, and available resources, shared mechanisms treatments have found a wide range of applications. However, their use in school-based delivery modes may be limited due to the specific context and resources available [49].

##### Cognitive behavioral therapy (CBT)

Cognitive Behavioral Therapy (CBT) is a well-established and widely used transdiagnostic treatment approach, which targets shared mechanisms across various mental disorders [28]. CBT is based on the cognitive model, which posits that maladaptive thought patterns contribute to the development and maintenance of psychological disorders. By identifying and challenging these thought patterns, CBT aims to alleviate symptoms and improve functioning across a range of disorders [50].

CBT has been adapted for use in various formats, including individual, group, and internet-delivered therapy. Internet-delivered CBT (i-CBT) has emerged as a promising approach to increase the accessibility of CBT, particularly for individuals who may face barriers to in-person treatment. For instance, a recent study found that guided i-CBT was effective in treating anxiety and depression among university students, demonstrating the potential of i-CBT as a scalable and cost-effective treatment option [51].

Despite its broad applicability, CBT is not a "one size fits all" approach. The effectiveness of CBT can vary depending on the specific disorder, the individual's characteristics, and the context in which it is delivered. Therefore, ongoing research is needed to optimize the delivery of CBT and maximize its benefits across diverse populations and settings.

### Potential benefits of transdiagnostic approach

Transdiagnostic approaches, though not universally defined, were conceived as a response to the constraints of traditional diagnostic systems such as the DSM and ICD. These approaches aim to treat mental disorders from a broader perspective, considering higher dimensions and addressing multiple co-occurring conditions [52]. Transdiagnostic approaches have shown promise in treating mental disorders holistically [53]. While they may not produce exceptionally prominent effects on specific disorders or symptoms in individuals with co-occurring conditions, they tend to be more effective than single-diagnosis interventions in reducing a broader range of mental issues, without focusing solely on clinical diagnoses or symptoms [54].

Transdiagnostic approaches are also in line with the current trend of expanding the scope of mental health. It is increasingly recognized that good mental health and mental disorders are not simply two ends of a continuum among children and adolescents [55]. The conventional perspective, which equates the absence of mental disorders with good mental health, stems from the dominance of medical, disease-centered approaches in psychiatric research over the past decades [56]. However, researchers now acknowledge that positive mental health encompasses more than the absence of mental disorders [15]. The World Health Organization (WHO) defines mental health as a state of well-being in which individuals realize their abilities, cope with normal life stresses, work productively, and contribute to their communities. This definition explicitly acknowledges the importance of factors beyond the absence of mental disorders [57]. Consequently, researchers are expanding their perspective on mental health promotion to encompass a broader scope, moving beyond a sole focus on preventing mental disorders. This paradigm shift is accompanied by a notable increase in acceptance and recognition of interventions aimed at promoting mental health in children and adolescents from a comprehensive standpoint [58]. This trend of scope expansion aligns with the development of the transdiagnostic concept.

Given the possibility that previous literature may have been primarily influenced by a traditional viewpoint, which predominantly emphasized mental health promotion programs as a means of preventing mental disorders, it is crucial to adopt a more inclusive perspective that embraces the broader aspects discussed earlier. To address this need, the current study aims to examine relevant literature by categorizing the included studies into two distinct groups: those that explicitly emphasize the prevention of mental disorders and those that take a comprehensive approach by promoting the overall mental health of children and adolescents. Employing this classification framework allows for a comparison between studies reflecting the two viewpoints, thereby facilitating a more nuanced understanding of the diverse interventions and their respective objectives.

### The role of conductor

The school environment plays a crucial role in effectively improving the mental health of children and adolescents at a collective level, making it a promising setting for implementing universal transdiagnostic interventions. Within this context, the involvement of teachers in school-based projects appears to be inevitable, regardless of whether they serve as conductors or participants in the intervention. When teachers assume the conductor role, transdiagnostic protocols have shown to be highly effective. These interventions enable conductors to work on shared mechanisms of multiple diagnoses and influential factors to promote general mental health, such as resilience, self-esteem, and social and emotional skills [59]. Many of these elements have been well-validated or adapted from classic single-diagnosis protocols, such as Cognitive-Behavioral Therapy (CBT), making them more accessible to teachers who may not be mental health professionals.

From an economic perspective, the school and education sector shoulders approximately 90% of the general cost related to the mental health and emotional well-being of children and adolescents [60]. In this regard, utilizing teachers as conductors of school-based universal transdiagnostic programs for promoting mental health can help reduce the economic burden compared to employing professional psychologists. Additionally, from the perspective of students, research conducted by the National Health Service (NHS) has indicated a preference for seeking help from teachers when dealing with mental health issues (NHS Digital, 2018). Furthermore, since students spend a significant amount of time with teachers, they may have a better understanding of and ability to address potential negative impacts of other social factors experienced by students [61, 62].

Despite the scattered evidence supporting teachers as conductors, there has been no comprehensive systematic review conducted to examine whether teachers or psychologists are better suited as conductors for school-based universal transdiagnostic interventions aimed at promoting the mental health of children and adolescents. Therefore, it is essential to conduct a systematic review that comprehensively evaluates this question, considering the potential benefits and limitations associated with each approach.

While this review seeks to uncover the relative effectiveness of teacher-led versus psychologist-led transdiagnostic interventions in schools, it is pertinent to acknowledge existing research that sheds light on the influence of instructors' professional backgrounds on intervention outcomes. Studies such as Werner-Seidler et al. [13] and van Loon et al. [12] have conducted moderator analyses to explore this very question within the broader context of mental health interventions. For instance, Werner-Seidler et al. [13] found that interventions delivered by professionals were generally more effective than those conducted by school personnel, suggesting that the expertise and training of the conductor can significantly impact the success of the intervention. These findings, although not directly focused on universal transdiagnostic approaches, underscore the importance of considering the instructor's role in the design and implementation of school-based mental health programs. As such, insights from these studies are invaluable to our investigation, providing a foundation upon which to explore the comparative efficacy of teachers and psychologists in delivering universal school-based transdiagnostic interventions.

### Rationale

The development of transdiagnostic theory and applications has significantly contributed to the emergence of universal school-based transdiagnostic interventions as a novel approach for promoting the mental health and emotional well-being of children and adolescents. Although several studies have utilized validated frameworks such as mindfulness, FRIENDS (a cognitive behaviour therapy (CBT) based programs), and acceptance and commitment therapy (ACT) within this context [63–66], the potential benefits of transdiagnostic approaches in mental health promotion within a universal school-based context have not been systematically examined.

Furthermore, there is an evolving diversity of delivery modes for school-based universal approaches to mental health promotion [8]. These approaches can be class-based, delivering a curriculum, or they can focus on creating a positive environmental ethos at the whole school level by addressing aspects such as teacher discipline practices and reducing bullying. In those cases, the programs' participants may go beyond students and involve parents and teachers as long as the programs are conducted within the school environment. At the same time, school identification is not based on academic or instructional criteria but instead on the age of the students where it involves, considering the present study aims to examine the programs scoping at children and adolescents’ mental health. In this case, tertiary education institutions may also be involved, as the present study takes ages 10–19, defined as adolescence by WHO. Additionally, some universal approaches aim to improve the mental health atmosphere at both class and school levels. The choice of delivery mode and the individuals involved in delivering the interventions, whether teachers or psychologists, may influence the efficacy of transdiagnostic approaches in promoting the mental health and emotional well-being of children and adolescents [15]. Therefore, it is important to examine the influence of delivery mode and delivery persons in a systematic review of school-based universal transdiagnostic promotion programs.

Moreover, many studies evaluating school-based universal transdiagnostic interventions do not clearly identify themselves as transdiagnostic, even if they employ transdiagnostic protocols or practices. Additionally, studies focusing on universal transdiagnostic approaches in the school context are often unsystematic in various aspects, such as the application of transdiagnostic theoretical frameworks, leading to potential limitations in the quality of research. This is particularly relevant for school-based universal transdiagnostic interventions aimed at promoting the mental health and emotional well-being of children and adolescents. Therefore, it is crucial to conduct a systematic review to assess the research quality of school-based universal transdiagnostic interventions and provide insights on how to enhance the validity and reliability of these interventions from a research perspective. The present study represents a significant contribution as it will be the first systematic review explicitly focusing on transdiagnostic interventions for promoting children and adolescents' mental health and emotional well-being. This review distinguishes itself from others in two key aspects: firstly, it aims to identify and evaluate transdiagnostic programs explicitly, and secondly, it encompasses programs applicable to any age range within the definition of children and adolescents. While some recent systematic reviews may cover similar programs, it is important to note that certain programs adhere to transdiagnostic principles without explicitly identifying themselves as such, and they may also target the age range of similar interest but limited. For instance, Fenwick-Smith et al. [31] and Sutan et al. [67] may have some overlap with the programs under investigation. However, this study aims to provide a comprehensive analysis specifically focused on transdiagnostic interventions for children and adolescents, thereby filling a crucial research gap.

In light of these considerations, the present study aims to conduct a systematic review to provide reliable knowledge that can guide future studies and practices of school-based universal transdiagnostic approaches in promoting the mental health and emotional well-being of children and adolescents. The following research questions, derived from previous studies, will be critically examined:

1. How effective are universal school-based transdiagnostic interventions in promoting the mental health of children and adolescents, and what methodologies have been employed in these interventions?
2. Are there differences in the effectiveness of interventions with the goal of promoting mental health compared to those aimed at preventing mental disorders?
3. Are there any differences in outcomes between teacher-led and psychologist-led universal school-based transdiagnostic interventions?

## Methodology

### Protocol

The present systematic review was conducted in accordance with the Preferred Reporting Items for Systematic Reviews and Meta-Analyses (PRISMA) statement [68]. The protocol was developed based on the Cochrane Handbook for systematic reviews [69].

### Searching strategy

To identify relevant studies, comprehensive systematic searches were undertaken on the three databases, psycInfo, pubmed, and web of science by medical subject headings (MeSH) and text word terms: (Universal or whole-school or school-wide) AND ("mental health" OR "mental health intervention" OR "early intervention") AND (school-based OR (scho* OR educat*)) AND (Adolescen* OR child* OR teen* OR youth OR young peop* OR pupil* OR student* OR learner* OR scho*) AND (Anxi* OR depress* OR resilien* OR emotion* OR stress* OR psycho* OR wellbeing* OR prevent* OR promot*) AND (Evaluation OR (pilot OR trial) OR (comparison OR effective)) AND (treatment or psychotherapy OR "cognitive therapy" OR psychotherapy OR brief OR rational-emotive or therapy OR "treatment outcome" OR "implosive therapy" OR "exposure therapy" OR "cognitive behavioural therapy" OR "cognitive behaviour therapy" OR "cognitive behavior therapy" OR CBT OR "acceptance and commitment therapy" OR ACT OR MBCT OR "mindfulness based cognitive therapy" OR "mindfulness treatments" OR mindfulness OR "mindfulness-based stress reduction" OR "dialectical behaviour therapy" OR DBT OR "meta-cognitive therapy" OR "psychotherapy outcome" OR "unified protocol" OR UP OR "transdiagnostic therapy" OR "interpersonal therapy" OR IPT OR “Shaping Healthy Minds” OR “MATCH Protocol” OR MATCH-ADTC OR counselling OR counseling OR transdiagnostic).

The studies were included if they are qualified with the following criteria: (a) they are transdiagnostic interventions. The selection of transdiagnostic intervention followed the definition of transdiagnostic intervention posited in the literature review section, with no restriction upon the emergence of the word “transdiagnostic” in the studies. However, an explicit meta-identification under transdiagnostic framework is mandatory. (b) They are school-based. The recipients are not necessarily students. If the direct recipients are teachers or parents, through whom the effect of the intervention applied on the children or adolescents should be presented. (c) They are conducted with participants (aged over four years old) without particular holistic psychiatric features (universal). (d) They aim to promote mental health and/or emotional wellbeing. Following the justification in the literature review, preventative measures are also taken as a type of mental health promotion. (e) They are written in English; (f) published in a peer-reviewed journal.

The articles that are written in languages other than English and not peer-reviewed journals were excluded. Studies without pre-post tests were also excluded. The articles without clear frameworks or conceptual basis of interventions that make them identifiable transdiagnostic were also excluded. Studies with participants’ current student status over college-level or age over 24 were excluded. Studies employing pure qualitative measurement for mental health were excluded. In addition, studies about indicated or selective interventions were not included.

### Identification and screening process

**Fig. 1:**
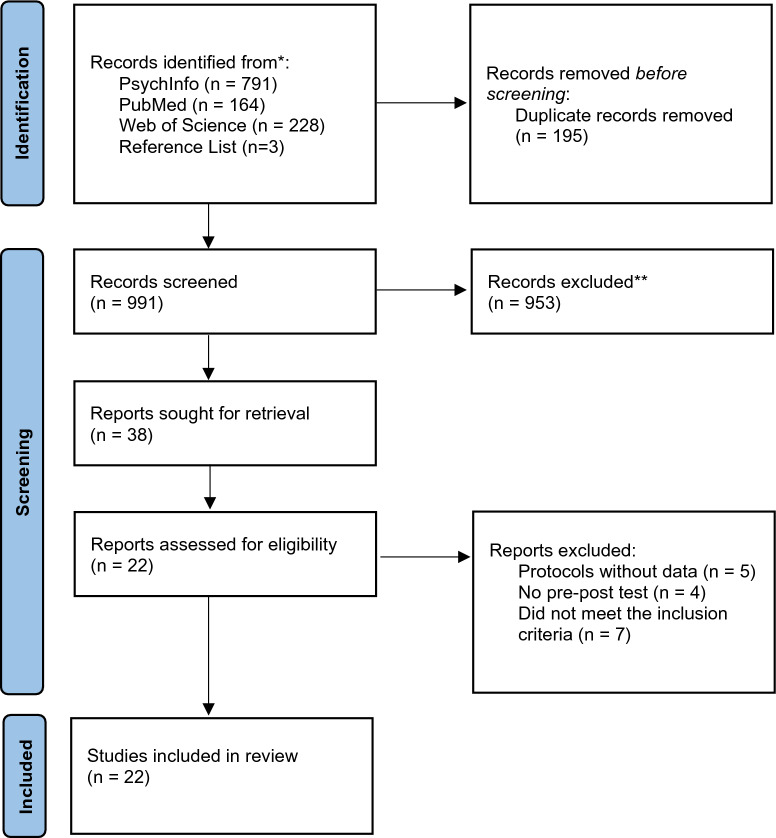
PRISMA flow diagram illustrating the selection process

In the first stage, the search was completed, and the articles retrieved were managed with Endnote. In the second stage, after removing the duplicates, one researcher scanned the titles and abstracts of the 991 studies against the inclusion and exclusion criteria to determine their relevance to the current research. The studies with titles and abstracts that are evidently irrelevant were first discarded, and 38 articles were passed to the next stage. At stage three, the full text of the retrieved articles through this process was also examined against the following criteria. Twenty-two studies were finally included for review. At each stage, 10% of the articles that passed the initial scanning were evaluated by another researcher. The agreement was reached after a critical discussion. The whole process following the PRISMA guidelines is illustrated in Fig. 1.

### Quality assessment

The present review employed Downs and Black Checklist [70] to conduct the quality assessment. This checklist includes 27 items, assessing the internal and external validity, study power, and selection bias. This checklist was chosen mainly for its’ feasibility in public health relevant studies across randomized and non-randomized designs.

Another researcher has assessed a 10% sample of studies after the first evaluation covering all shortlisted conducted by the main researcher, with only one study disagreed. Potential rating discrepancy was solved through discussion until reaching a shared decision. Considering that one of the important research purposes is to evaluate the efficacy of the school-based transdiagnostic approach in mental health and emotional wellbeing promotion, any study with poor quality was excluded to prevent a potential negative influence on study quality. The quality rating of included studies was presented in Table 1.

**Table 1:** Intervention characteristics

Study quality	Study design	Measures	Follow-up	Conductor	Intervention	Effects/Outcomes	General study aim	School level
Berry et al. [71] (68.8%)	Cluster Randomized Control Trial; Randomized on a 1:1 ratio stratified with binary classification of school size and eligible children proportion for free school meal; 29 schools "PATHS" intervention × 27 schools WL control	SDQ PTRS T-POT TBQ	Baseline, 1-year follow-up, 2-year follow-up,	Teacher	SEL	12 months follow-up: No difference on SDQ, some significant differences on PTRS (social competence: d = 0.09, aggressive behaviour:d = − 0.14,inattention: d = − 0.06, Impulsivity: d = − 0.06, peer relations: d = − 0.10, learning behaviours: d = 0.10); 6 months follow-up: Significant difference on T-POT (Total teacher positive behaviours: d = − 0.304, Class negative behaviour to teacher: d = 0.307, Class off-task behaviour:d = 0.227)	Promotion	Primary school
Bradshaw et al. 2013 (68.8)	Open cohort 4-year RCT:new students to intervention school can participants while those who left intervention schools would not be traced; 21 schools randomized to intervention group, 16 schools randomized to control group	TOCA-C	5 times of evaluation over the four years: Baseline in the fall of the first year, and follow-up in the spring of the following four years.No particular time point reported	Teacher	CBT	Significant effect on the general sample: disruptive behaviors (d = 0.12), concentration problems (d = 0.08), prosocial behavior (d = − 0.17), emotion regulation (d = − 0.11), IG children are 33% likely to receive an ODR (AOR = 0.67),IG girls less likely to receive an ODR than CG girls (AOR = 1.27); Significant effect on the participants who are at kindergarten at the onset of the trial than CG: prosocial behavior (γ = 0.08), emotion regulation (γ = 0.05); no significant differences between SWPBIS and comparison schools on suspensions	Promotion	Elementary school
Burckhardt et al. [72] (43.4%)	Quasi-randomised controlled trial with cluster randomization. 1: 1 ratio of intervention and control group number (n = 4);disproportionate participants number to intervention (n = 63) and control group (n = 61).After follow-up, intervention was also delivered to the control group for evaluation	DASS-21 FS	Baseline, Post-intervention, 5-month follow-up	Teacher	ACT	No significant difference between IG and CG on baseline;no significant differences between post-intervention completers and dropouts;no differences were found between follow-up dropouts and completers.No significant differences between the ACT and control conditions for the DASS-21 or FS on different time points.Baseline-post effect size on (non-significant): depression: d = 0.31, anxiety: d = 0.28, stress: d = 0.63; DASS-total: 0.44, FS: d = 0.20. General positive effect of ACT reported by both groups: agreeing over 50%, except exercising more	Prevention	High school
Cook et al. [73] (34%)	A matched quasi-randomized design was used to equate groups at baseline. A quasi-experimental procedure and not pure random assignment were conducted, producing four pairs of classes were assigned to differentt experimental conditions	SIBS SEBS	Baseline, Post-test (5 months after baseline collection)	Teacher	SEL and PBIS	Externalizing: SEL Only (d = − 0.24), PBIS Only (d = − 0.26), SEL/PBIS COMBO (d = − 0.62), Control (d = 0.00); Internalizing: SEL Only (d = − 0.16), PBIS Only (d = − 0.02), SEL/PBIS COMBO (d = − 0.37), Control (d = 0.00). The significance of all data was not reported	Promotion	Elementary school
Dowling et al. [74] (59%)	Cluster Randomized Control Trial; 1: 1 ratio of intervention and control group number (n = 17);disproportionate participants number to intervention (n = 345) and control group (n = 330)	RSS ERQ TMMS CSI-15 SEQC AICQ MDELS DASS-21. WEMWBS ATSS SAMRS	Baseline, Post-intervention	Teacher	SEL	Significant increase of Social Support Copings (d = 0.15), Significant reduction of suppressing emotions (d = − 0.19), Significant reduction of stress (d = − 0.06) and depression (d = − 0.07)	Promotion	High school
Dvořáková et al. [75] (50%)	Pilot randomized control trial. Gender stratification was conducted in the randomization to assure a balanced gender ratio in the intervention (n = 55, n of female = 35) and control (n = 54, n of female = 37)	GAD SWL MAAS SCS SCC-R. CS PSQI YAAPST	Baseline, post-intervention	Psychologist	Mindfulenesss	Significant effect on anxiety (d = − 0.48), depression (d = − 0.34), and satisfaction with life (d = 0.41)	Promotion	University
Flynn et al. [76] (37.5%)	Quasi-experimental design with opportunistic sampling. No blinding or randomization was reported. DBT STEPS-A (n = 26) × control group (n = 45)	DBT-WCCL. BASC-2	Baseline, post-intervention	Teacher	SEL	Significant effect on emotion symptom index (d = − 0.32) and internalizing (d = − 0.41)	Promotion	All-female high school
Garcia-Escalera et al. 2020 (46.9%)	Two-arm cluster randomized control trial. UP-A group (n = 90) × waitline control group (n = 61)	RCADS-30 CDN EAN DPDSQ CKQ SPOQ	Baseline, postintervenion, 3-months follow-up	Psychologist	UP	Significant main effect of time on the RCADS total score (d = − 0.22), Separation Anxiety Disorder (SAD, d = − 0.32), Generalized Anxiety Disorder (GAD, d = − 0.18), Obsessive Compulsive Disorder (OCD, d = − 0.21). Significant decreases for those with severe baseline symptoms in the UP-A group between the following time points: T1 and T3 (d = 0.96, n = 9) and T2 and T3 (d = 0.88, n = 9)	Prevention	High school
Johnson and Wade, 2019 (56.3%)	Cluster (class based) controlled trial. Mindfulness intervention group (n = 71) × Control group (n = 75)	DASS-21 GAD-7 EDE-Q. WEMWBS	Baseline, post-intervention, 4-months follow-up	Teacher	Mindfulenesss	No significant effect was reported on post-intervention. High medium significant effect reported on the anxiety (d = 52) and depression (0.61) among all intervention group students in follow-up. Moderation effect: only significant effect on anxiety (d = 0.81) and depression (d = 0.95) among year ten students	Promotion	Secondary school
Johnson and Wade, [63] (59%)	Cluster (class based) controlled trial. Mindfulness intervention group (n = 237) × Control group (n = 239)	CHIME-A. DASS-21 GAD-7 EDE-Q. WEMWBS	Baseline, post-intervention, 3-months follow-up, 9-months follow-up	Teacher	Mindfuleness	Significant effect on Decentering and Nonreactivity (d = − 0.20) among the general sample in 3 month follow-up. Year 8 at 3 month follow-up: Awareness of External Experiences (d = − 0.30), Decentering and Nonreactivity (d = − 0.39), wellbeing (d = − 0.25)	Promotion	Secondary
Kishida et al. [77] (81.3%)	No randomization was conducted	JSDQ GSESC_x005f-R Short CAS. DSRS-C ASCA	Baseline, post-intervention	Teacher	CBT	Study 1: Significant time effects for general difficulties [F (1, 52.78) = 4.88, p < 0.05], general difficulties (SDQ) decreased with small effect sizes (g = − 0.20). For subclinical samples in study 1, no significant time effects for the primary and secondary outcomes; general difficulties, internalizing problems, and externalizing problems decreased with medium effect sizes (g = − 0.79, g = − 0.52, and g = − 0.60, respectively). Study 2: no significant time effects or meaningful effect sizes for all outcomes. subclinical sample: Significant time effects for depression as a secondary outcome [F (1, 9) = 6.45, p < 0.05], General difficulties and internalizing problems, anxiety, and depression decreased with medium effect sizes (g = − 0.69, g = − 0.55, g = − 0.63, and g = − 0.63, respectively)	Prevention	Primary
Knight et al. [78] (75%)	Conductor (teacher) randomization, and student control trials. Teachers aligned to different classes in the first year. In the second year, students may be alligned with different teachers and classmates, while they will remain in the same group (intervention or control)	SEARS	Baseline, post-intervention	Teacher	CBT and SEL	Significant effect on Self-regulation (d = 1.04), Social competence (d = 0.65), Empathy (d = 0.70), Responsibility (d = 0.81), SEARS-T (d = 0.89). No significant effect reported on SEARS-C/A	Promotion	Secondary
Kuyken et al. [64] (50%)	Non-randomised controlled parallel group study. MiSP group (n = 256) × Control group (n = 266)	WEMWBS PSS CES-D	Baseline, post-intervention, 3 months follow-up	Teacher and psychologist	Mindfulness	Significant fewer depressive symptoms post-treatment and at follow-up and lower stress and greater well-being at follow-up	Promotion	Secondary
Lam and Seiden [79] (34%)	Controlled trial, no randomization was reported	BRIEF-SR DERS RRS	Baseline, post-intervention	Psychologist	ACT, SEL, and Mindfulness	Significant effect on general YSR (η^2^ = 0.05) and EF (η^2^ = 0.11) score, specifically on anxiety/depression (η^2^ = 0.09), emotion (η^2^ = 0.06), monitor (η^2^ = 0.06), and memory (η^2^ = 0.11)	Promotion	Primary
Mendelson et al. [80] (43.3%)	Randomized control trial. RAP group (n = 29) × Control group (n = 20)	SDQ ACES SCS SMFQ CCSC EAQ	Baseline, post-intervention	Psychologist and a community member	CBT and Mindfulness	Significant effect on SCS Dysregulation (d = 0.85), SCS Social Competence (d = 0.87), SCS Authority Acceptance (d = 0.69), ACES Academic Competence (d = 0.76)	Prevention	Secondary
Sawyer et al. [81] (78.1%)	Cluster randomized controlled trial. Intervention group (n = 3037) × Control group (n = 2597)	CES-D OTSS AICQ CAS MSPS APSC. Interpersonal Competence Questionnaire, Coping Actions Scale. School-level protective and risk factors:Multidimensional Scale of Perceived Support, Adolescents’ perceptions of school climate	Baseline, 1-year follow-up, 2-year follow-up	Not reported	CBT	Multi-level modelling indicated significant effect on both intervention and control groups: CES-D scores (coefficient = 0.517, SE = 0.222), Positive Coping Strategies (coefficient = -0.496, SE = 0.147), Negative Coping Strategies (coefficient = − 0.248, SE = 0.100), Optimistic Thinking Style (coefficient = − 0.716, SE = 0.115), Family Relationships (coefficient = − 0.838, SE = 0.068), Friends Relationships (coefficient = − 0.188, SE = 0.077), Significant Other Relationships (coefficient = − .178, SE = 0.063), School Climate (coefficient = − 1.849, SE = 0.247); Significant coefficient for the group × time rating on teacher rated school climate (coefficient = 0.600, SE = 0.290). Students with mild to severe CES-D baseline scores: reduction in Negative Coping Strategies (coefficient = − 0.708, SE = 0.129), Relationships (coefficient = − 0.765, SE = 0.117) and student-rated School Climate (coefficient = − 1.392, SE = 0.252). Significant increase in Interpersonal Competence (coefficient = 0.352, SE = 0.164)	Promotion	Secondary
Shum et al. [82] (50%)	Non-randomized controlled trial. Intervention group (n = 264) × Control group (n = 195)	SCARED ATS-N/P. C-IRI RSES	Baseline, post-intervention, 6-months follow-up	Students	CBT	Significant effect on the knowledge of mental health in post-intervention (coefficients = 0.46) and follow-up (coefficients = 0.66), and perspective-taking (empathy, coefficients = 1.50). Online program: compared with lower completion group, significant improvement on higher completion group on the knowledge of mental health (coefficients = 0.51) and positive thoughts (coefficients = 3.32)	Prevention and promotion	Primary
Skryabina et al. [83] (59%)	Cluster randomized control trial. Health-led FRIENDS (n = 478) × School-led FRIENDS (n = 467) × Usual PSHE (n = 398)	RCADS PSWQC RSES	Baseline, 12 months follow-up	Teacher and psychologist respectively	CBT	Significant reductin on Sep anxiety: Health-led FRIENDS group × Usual PSHE group (d = − 0.11), School-led FRIENDS group × Usual PSHE group (d = − 0.17)	Prevention and promotion	Primary
Stallard et al. [65] (75%)	Cluster randomised controlled trial. Health-led FRIENDS (n = 509) × School-led FRIENDS (n = 497) × Usual PSHE (n = 442)	RCADS-30. PSWQC RSC OBVQ SDQ RCADS-30-P	Baseline, 6-months follow-up, 12-months follow-up	Teacher and psychologist respectively	CBT	All groups: Significant difference in the mean of RCADS at 12 month for health-led FRIENDS, comparing school-led FRIENDS (interaction coefficient − 3.91) and usual school provision (interaction coefficient = − 2.66). High risk group: signficant 12 month within group reduction, without effect difference across groups. Low risk group: Significant difference in the mean of RCADS at 12 month for health-led FRIENDS, comparing school-led FRIENDS ((adjusted difference = − 3.78) and usual school provision (adjusted difference = − 3.13). Low anxiety group: significant effect on health-led FRIENDS, than usual school provision (d = 0.22) and school-led FRIENDS (d = 0.25), reflected by child-completed total RCADS	Prevention	Primary
Takahashi et al. [66] (56.3%)	Non-randomized controlled trial. Intervention group (n = 69) × Control group (n = 230), alligned on teacher's decision	VOYAGE, SDQ	Baseline, post-intervention	psychologist	ACT	Significant group × time interaction effect on Continuation of Avoidance subscale (CA, β = − 0.97), the ACT group showed significant reductions in CA between pre- and post-intervention periods (β = 0.86). Significant reduction in CA in ACT group between pre- and post-intervention periods (β = 2.02), while not in the WLC group (β = − 0.58). Significant interaction effect for the Hyperactivity/Inattention subscale on the SDQ in the whole sample (β = − 0.69). Both WLC (β = 0.22) and ACT group (β = 0.92) showed significant reduction in Hyperactivity/Inattention	Promotion	Secondary
Torok et al. [84] (34%)	Non-experimental pilot examination with pre and post-test	Teacher-rated SDQ	Baseline, post-intervention	Teacher	SEL	Significant effect on SDQ global difficulties (d = 0.44), Externalising difficulties (d = 0.30), Internalising difficulties (d = 0.36), Emotional (d = 0.30), Conduct (d = 0.21), Hyperactivity (d = 0.29), Peer relationships (d = 0.30), Prosocial behaviour (d = 0.26)	Promotion	Primary
Volkaert et al. [85] (68.8%)	Cluster randomized control trial, without blinding participants. Intervention group (n = 139) × Control group (n = 208)	DERS FEEL-KJ. SPPA HRQoL CDI CESD STAI-C SSRPH OBVQ ARSP CBCL BRIEF	Baseline, post-intervention, 3-month follow-up, 6-month follow-up	Psychologist	Locally Designed Transdiagnostic	Significant group × time interaction effect on depressive symptoms***, CDI*, anxiety symptoms***, negative affect***, self-esteem***, quality of life***	Promotion	Primary

### Data extraction

This study extracted the research purpose, information of intervention (framework, duration, and delivery details), sample features, procedure, measurement, and outcome/result. In addition, the implementation was emphatically examined to analyze the influence of school-based delivery.

### Synthesis

The major aim of the present study is to expose the effectiveness of universal school-based transdiagnostic interventions in promoting children and adolescents’ mental health. The outcomes of the included studies were interpreted following Cohen [86] and Hedges 87, taking small (d = 0.2; g = 0.2), medium (d = 0.5; g = 0.5), and large (d = 0.8; g = 0.8). Some studies did not report full descriptive data; therefore, the original multilevel modeling result as coefficients was also taken [65, 81, 82]. Due to the heterogeneity, the specific outcomes were spelled out with narratives. For the convenience of synthesis, most of the outcomes were displayed in effect size. Some of the studies’ outcomes were demonstrated in standardized (interaction) coefficient reflected by multilevel modeling as expressed in the original articles because of the lack of relevant descriptive data to conduct secondary calculations. Only the statistically significant effects were reported.

## Results

### Study selection

The database search has offered a total of 1183 results. After removing duplicates, screening titles, abstracts, and full text against the inclusion and exclusion criteria, and a manual search of the reference list for eligible articles, 23 articles were finally involved in the review.

### Data extraction

#### Overview

Of the 23 articles sourced, 1 took place in both early years and primary education, 9 in primary education, 11 in secondary education, 1 in higher education (university).

#### Intervention characteristics

The interventions included in this study exhibit a range of objectives related to promoting the mental health of children and adolescents. The majority of interventions (n = 15) have a general aim of enhancing mental health outcomes, while some interventions (n = 5) specifically focus on the prevention of mental disorders, particularly anxiety and depressive symptoms. Additionally, two studies aim to achieve both prevention of mental disorders and the promotion of protective factors for mental health. It is worth noting that all of the interventions, either implicitly or explicitly, utilize transdiagnostic protocols. Table 1 provides a comprehensive overview of the key characteristics of these interventions, including their objectives. For a more detailed analysis, the appendixes will contain additional information such as participants' demographic data, theoretical basis, intervention delivery methods, and fidelity.

### Interventions to promote

Among the interventions aimed at promoting the mental health of children and adolescents, there were several distinct approaches. Specifically, seven interventions focused on improving social and emotional learning (SEL) capabilities, which encompassed foundational skills like emotion regulation (ER) and interpersonal problem-solving [71, 73, 74, 76, 78, 79, 84]. All four studies that solely employed SEL principles were teacher-led interventions. In contrast, only one intervention was integrated into the regular school curriculum [74], while the remaining three utilized various forms of delivery, such as physical exercise, group discussions, and home tasks. Flynn et al. [76] implemented DBT-STEPS-A, based on dialectical behavior therapy (DBT) theory, and conducted three assessments to evaluate the targeted emotional skills.

Five interventions utilized mindfulness-based approaches to enhance protective factors and reduce risk factors, such as stress reduction ability, resilience, and emotional skills [63, 64, 75, 79, 88]. Among the four mindfulness-based interventions, three were teacher-led, while psychologists led the remaining one. L2B by Dvořáková et al. [75] differed from the other interventions as it was psychologist-led and targeted first-year undergraduates, who were predominantly within the age range of adolescents according to WHO definitions. Additionally, L2B had a longer duration of sessions, totaling approximately 640 min, which was longer than most other interventions. Kuyken et al. [64] did not provide specific details about the intervention length.

One intervention was based on Acceptance and Commitment Therapy (ACT) principles, while another intervention utilized a locally-designed transdiagnostic theory that emphasized acceptance of adverse situations [66, 85]. The ACT intervention focused on dedicating oneself to the present moment after accepting negative experiences, whereas the locally-designed transdiagnostic intervention placed greater emphasis on the ability to regulate emotions during challenging experiences. The ACT intervention comprised lecture-based sessions held bi-weekly over four months and was led by psychologists. The locally-designed transdiagnostic intervention consisted of a two-day program followed by a half-day booster session before each follow-up and was also led by psychologists.

Two interventions solely employed Cognitive-Behavioral Therapy (CBT) principles, targeting both students and teachers. These interventions were integrated into the regular school curriculum and aimed to promote positive thinking and behaviors, enhance protective factors, reduce risk factors, and foster a positive school ethos to collectively support students' mental health (Bradshaw et al. 2013; [81]). Both interventions were teacher-led.

Three interventions integrated multiple principles, including SEL, CBT, and mindfulness, sharing characteristics mentioned above. Among them, Cook et al. [73] introduced Positive Behavioral Interventions and Supports (PBIS), a new universal practice led by teachers, in addition to SEL, to enhance the school system's support for students' mental health. The other two interventions, SEL@MS by Knight et al. [78] and L2B by Lam and Seiden [79], incorporated features of CBT and ACT principles. SEL@MS was led by teachers, while L2B was led by psychologists. All three interventions employed various forms of delivery, such as lectures, group discussions, and mindfulness practices.

### Interventions to prevent

Among the interventions aimed at preventing mental disorders, several different approaches were employed. Two interventions were based on Cognitive-Behavioral Therapy (CBT) principles: Up2-D2 by Kishida et al. [77] and FRIENDS by Stallard et al. [65]. Burckhardt et al. [72] implemented an intervention based on Acceptance and Commitment Therapy (ACT), while Garcia-Escalera et al. (2020) utilized the Unified Protocol (UP) approach in their intervention (UP-A). Mendelson et al. [80] developed the RAP Club intervention, which combined elements of CBT and mindfulness-based principles.

UP-A was led by psychologists, FRIENDS by Stallard et al. [65] was conducted in both teacher-led and psychologist-led contexts to compare the efficacy influenced by different facilitators, and RAP Club was implemented by psychologists as well as community members. The remaining interventions were all teacher-led. All of these interventions aimed to prevent anxiety, depression, or both, recognizing mental disorders or symptoms as integral components of mental health promotion.

### Interventions with mixed aims

The two interventions with mixed aims focus on preventing anxiety disorders while also promoting students' emotional skills. One intervention, The Adventures of DoReMiFa by Shum et al. [82], is self-conducted by students and is based on Cognitive-Behavioral Therapy (CBT) principles. It employs a combination of digital game-based and school-based settings. The intervention aims to enhance students' social and emotional capabilities through the use of digital games, which are further reinforced through school-based lectures.

The second intervention, FRIENDS by Skryabina et al. [83], is teacher-led and also based on CBT principles. Although it was originally designed to solely prevent anxiety disorders, it also aims to promote other aspects of students' mental health, such as subjective well-being (SWB). This intervention takes a comprehensive approach to address multiple dimensions of mental health alongside anxiety prevention. It emphasizes the development of emotional skills to enhance students' overall well-being.

### Methodological quality

The quality of the included studies varied, with some studies rated as "poor" (34% for [73], [79], 37.5% for [76]) and others rated as "excellent" (75% for [78], 81.3% for [77]), based on the assessment criteria used.

All of the studies utilized a pre-post design to evaluate the effectiveness of the interventions. Thirteen studies employed randomized controlled trials (RCTs), with nine of them using cluster randomized controlled trials and four utilizing randomization at the individual level. Nine studies did not employ randomization, and seven studies utilized controlled trials.

The characterization of participants' demographic information, particularly age data, was often inadequately described across most studies (detailed information provided in the appendix). Only a few studies aimed to explore the contextual factors by providing information about the schools and socioeconomic backgrounds in which the interventions were conducted [63, 72]. Additionally, there was limited information provided regarding the demographic characteristics of the teachers, despite many interventions being teacher-led. Only one study indicated the length of teachers' working experience in the education sector [84].

Sample sizes varied considerably, ranging from 58 participants [77] to 12,334 participants (Bradshaw et al. 2013). The age range of the participants generally fell between 4 and 19 years old (specific details in the appendix). While the majority of the included studies focused on the secondary education sector, it is important to note that many studies conducted in primary education targeted participants in the late-primary age range. Moreover, it is worth mentioning that most studies did not conduct a power analysis to determine an appropriate sample size that would ensure statistically significant results and valid effect sizes.

### Intervention effectiveness

The outcomes are also presented in Table 1.

#### Measurement of intervention outcomes

The included studies utilized a diverse range of measurements to assess the outcomes of the interventions. These measurements captured various aspects, including children and adolescents' behavioral, social, and emotional development, general mental health and emotional wellbeing, as well as anxiety and depressive disorders. Additionally, some studies also measured behavioral and emotional factors among teachers [71].

When examining the overall results, a general trend can be observed, indicating that studies with higher research quality tended to report lower and less positive effects. This observation aligns with findings reported by Mackenzie and Williams [9] and suggests that the rigor of study design and methodology may have an impact on the outcomes observed in the interventions.

#### Children and adolescents’ social, emotional, and behavioral development

Six studies utilized the Strengths and Difficulties Questionnaire (SDQ) to screen and evaluate emotional and behavioral problems among children and adolescents. Among these studies, only two reported statistically significant improvements, albeit of small to medium effect sizes, on the SDQ or its subscales.

For example, Kishida et al. [77] observed a significant small decrease in general difficulties (SDQ) in study 1 (Hedge's g = 0.2) and a significant medium decrease in study 2 (Hedge's g = 0.69). It is worth noting that study 1 provided teachers with 1.5 h of training and supervision by psychologists, while study 2 offered only video and paper materials, which are generally considered less effective. Additionally, the sample size in these studies was small, necessitating the use of Hedge's g instead of Cohen's d, as the former is more suitable for small samples.

In the study by Takahashi et al. [66], multilevel modeling results indicated a significant time × group interaction effect on the SDQ (β = − 0.69), suggesting a statistically significant improvement in the intervention group compared to the control group. It should be noted that although the study was school-based and involved participants from four schools, the intervention was only implemented discretely at the class level within each school, limiting the potential benefits of a positive psychological environment.

Six studies reported positive effects on the social aspects of mental health [71], Bradshaw et al. 2013; [78, 80, 81, 84]. Among these studies, only one aimed to prevent mental disorders [80], while the other five aimed to promote mental health in children and adolescents. These programs primarily targeted social competence, peer relationships, prosocial behaviors, and empathy, yielding effect sizes ranging from d = 0.09 to 0.87. Notably, all five promotion programs were teacher-led, whereas the delivery of the prevention program involved collaboration between psychologists and community members.

Regarding emotional aspects, shared mechanisms across diagnoses were predominantly measured, including specific behaviors, risk and protective factors. Seven studies reported significant positive effects on these shared mechanisms. These effects encompassed reductions in aggressive/disruptive behavior (d = 0.12–0.14, [71], Bradshaw et al. 2013), improvements in inattention/hyperactivity (d = 0.06–0.29, [66, 71, 84]), enhancements in dysregulation (d = 0.85, [80]), emotion regulation (d = 0.11, Bradshaw et al. 2013), reductions in stress and emotion suppression (d = 0.06, 0.19, [74]), and reductions in emotional symptoms (d = 0.30–0.32, [76, 84]). All of these studies aimed to promote mental health in children and adolescents, with three of them specifically evaluating these factors at the emotional level, targeting shared mechanisms [71], Bradshaw et al. 2013; [66]. The remaining four studies also assessed mental disorders, which will be discussed later.

#### Anxiety and depression disorders prevention

Seven studies demonstrated significant improvements in anxiety-related outcomes [65, 66, 72, 74, 77, 88, 89], with effect sizes ranging from small (d = 0.18) to high (d = 0.81). Among these studies, four aimed to prevent mental disorders. Notably, Skryabina et al. [83] examined the effectiveness of the FRIENDS intervention in both teacher-led and psychologist-led groups and found a slightly larger but significant effect size in the teacher-led group (d = 0.17) compared to the psychologist-led group (d = 0.11) when compared with the control group. This study shared a similar design and intervention (FRIENDS) with Stallard et al. [65], which was also reviewed in Mackenzie and Williams [9]. However, unlike the interpretation of Stallard et al. [65] in Mackenzie and Williams [9] that fidelity to the intervention is positively associated with better outcomes, Skryabina et al. [83] indicated a similar fidelity rate between the groups but a larger effect size in the teacher-led (school-led) group, suggesting the potential existence of a confounding variable between fidelity and conductor identity.

Six studies reported significant improvements in depression-related outcomes, with effect sizes ranging from small (d = 0.07) to high (d = 0.63). Two of these studies aimed to prevent mental disorders. It is important to note that most of the effect sizes reported for depression were medium or high, except for Dowling et al. [74], which reported extremely small effect sizes (d = 0.07). Additionally, the study by Dowling et al. [74], which focused on high school students, appeared to be an outlier in terms of its overall significant outcomes, as all of the variables it reported had very small effect sizes.

#### Teacher behaviour and school ethos

Berry et al. [71] evaluated teacher-student interaction using the Teacher–Pupil Observation Tool (T-POT) and found significant improvements in teacher positive behavior (d = 0.31) and class negative behavior toward the teacher (d = 0.31). Although this aspect was not the primary focus of the study, the improved interaction between teachers and students indirectly indicates enhancements in students' social and emotional development.

Regarding the improvement of school ethos in terms of children and adolescents' mental health and emotional wellbeing, there were two studies. Only Sawyer et al. [81] reported significant effects on the school ethos as reflected in multilevel modeling. They found a group × time rating on teacher-rated school climate (coefficient = 0.600, SE = 0.290) and student-rated school climate (coefficient = -1.392, SE = 0.252). However, it is worth noting that the evaluations by teachers and students showed contradictory results in this study.

### Fidelity

Fidelity measurements were reported in the appendix of the studies. Some studies employed self-rated measurements [65, 71, 72, 74, 75, 77, 78, 81, 83, 84, 88], while others used external ratings [90]. However, it is important to note that many studies did not report the methods used for fidelity rating.

Due to the high heterogeneity of the studies, the relationship between fidelity and outcomes is not clear. As mentioned earlier, this relationship may be simply linear, but further investigation is needed to establish a clearer understanding.

## Discussion

This systematic review represents the first comprehensive examination of the effectiveness of universal school-based transdiagnostic interventions in promoting the mental health and emotional well-being of children and adolescents. It aims to bridge the gap between mental health promotion and mental disorders prevention by considering studies that explicitly focus on either aspect and those that view mental disorders prevention as a component of mental health promotion. Additionally, the review explores the potential impact of the roles played by teachers and psychologists in the effectiveness of these interventions, given that they are the most commonly involved professionals in promoting the mental health of children and adolescents.

### How effective are universal school-based transdiagnostic interventions that promote mental health or emotional wellbeing?

Based on the reviewed articles in this study, the overall effectiveness of universal school-based transdiagnostic interventions in promoting the mental health of children and adolescents is modest, consistent with a previous review that did not specifically focus on transdiagnostic methods [9]. However, considering the broad scope of transdiagnostic interventions, the overall effectiveness is noteworthy, as more than half of the included studies reported significant positive outcomes across a wide range of risk/protective factors, socio-emotional domains, and mental health disorders. This suggests that transdiagnostic approaches have had a widespread impact on the mental health of children and adolescents, particularly in promoting protective factors such as social and emotional competence, self-esteem, and self-regulation. Furthermore, there appears to be a positive association between study quality and the breadth of effective outcomes.

However, it is important to note that the effect sizes of interventions claiming to promote mental health were generally smaller compared to those targeting the prevention of mental disorders. Studies with the aim of preventing mental disorders were more likely to demonstrate larger effect sizes, albeit on a narrower range of factors, primarily anxiety and depressive disorders. While quantitative comparisons between promotion and prevention programs were limited due to heterogeneity, merging these two types of universal school-based transdiagnostic interventions and making appropriate adjustments to serve both aims simultaneously could potentially enhance overall effectiveness, as demonstrated in Cook et al. [73].

From a methodological perspective, the field of universal school-based transdiagnostic interventions for promoting children and adolescents' mental health is still in its early stages of development. Only half of the included studies employed randomized controlled trial (RCT) designs, with many others being pilot studies. This indicates that most universal school-based transdiagnostic programs are still in the developmental phase and require further testing. Only a few studies have been conducted with large samples, while many pilot studies of newly developed or adjusted programs exhibit relatively low research quality. Additionally, power analysis was rarely conducted, resulting in underpowered pilot studies with small sample sizes.

The validity and reliability of outcome measurements in intervention studies are also questionable. Various measurements were used to evaluate students' mental health, but many of them lack previous validation from scientific studies. The compatibility between the measurements and the specific aspects of mental health targeted by the interventions may be weak, particularly when self-developed measurements were used without scientific validation. Furthermore, follow-up assessments of interventions were inadequate in most studies. The majority only conducted pre- and post-tests, often with short-term post-test assessments. Only a small proportion of studies included multiple follow-ups with longer intervals. These studies revealed varying effects in the closest follow-up/post-test to the intervention and subsequent relapses in later follow-ups, suggesting potential near and far transfer effects. However, these effects were not further investigated in the studies, and the lack of extended follow-up assessments hindered a comprehensive understanding of intervention outcomes.

### The difference between teacher-led and psychologist-led interventions

Teacher-led interventions are the most common type of universal school-based interventions in promoting children and adolescents' mental health [91]. These interventions have been found to be more effective when applied to a large group of students. However, there are some concerns regarding teacher-led interventions highlighted in this review.

Firstly, as non-professional conductors, teachers require training to effectively deliver interventions. This training process can be time-consuming and financially burdensome, which may limit schools' willingness to adopt such interventions [76]. Moreover, the training of teachers is typically conducted by program developers or professional psychologists. This raises a potential confounding variable, as the students who receive the interventions may be exposed to secondary knowledge, despite their preference for teacher-led interventions. Regarding the training variable, one study included in this review compared psychologist-led training with supervision to self-conducted training using text and video resources. Surprisingly, the self-trained teachers achieved better outcomes when delivering the intervention [65].

Secondly, while interventions at the individual level are important, there is also a focus on school-wide effects and improving the general school ethos. In these cases, teachers have an advantage over psychologists due to their familiarity with the school system and class atmosphere [73]. However, among the studies included in this review, only two attempted to improve the school ethos to promote children and adolescents' mental health, and only one of them reported significant positive effects on the school environment [81]. Notably, the study that did not report significant effects on the school atmosphere was teacher-led, while the other study did not specify the identity of the conductors. Additionally, the studies reporting no significant effects were conducted on small samples, which may not fully represent schools. Therefore, it remains unclear whether psychologist-led or teacher-led delivery is more effective, as psychologists may have advantages in smaller sample sizes.

Thirdly, teacher-led delivery is more likely to be compatible with interventions embedded in the normal school curriculum. Only one study included in this review embedded the intervention in the regular school curriculum, and it was teacher-led [74]. While this single study may not be generalizable, it suggests that school curricula, especially at higher levels, are more lecture-based, which may lead to lecture-based interventions being embedded. However, most of the studies included in this review did not solely rely on lectures,instead, they utilized multiple forms of delivery, such as group discussions, physical exercises, and even digital games [82]. Therefore, the combination of teacher-led delivery and interventions embedded in the school curriculum may not necessarily align with the transdiagnostic approach.

### The scope of transdiagnostic frameworks

The studies included in the present review demonstrate a diverse range of theoretical frameworks. While traditional cognitive-behavioral therapy (CBT), mindfulness-based approaches, unified protocols (UP), and acceptance and commitment therapy (ACT) account for more than half of the interventions reviewed, there are also several studies that do not explicitly align with a specific theoretical framework. Instead, these studies focus on promoting children and adolescents' mental health through enhancing their social and emotional learning (SEL) in a transdiagnostic manner (e.g., [76, 79]).

Despite lacking a specific theoretical basis, these SEL-focused interventions have been shown to positively impact transdiagnostic constructs, particularly social and emotional factors, as mentioned earlier. As a result, these studies provide evidence supporting the acceptance of SEL interventions as a shared mechanism of transdiagnostic interventions. By recognizing the influence of social and emotional factors on various mental health conditions, the present review aims to promote the development of transdiagnostic theories.

In summary, while traditional CBT, mindfulness-based approaches, UP, and ACT are commonly utilized in transdiagnostic interventions, there is also a recognition of the importance of social and emotional learning as a transdiagnostic mechanism. The inclusion of studies focusing on SEL interventions without a specific theoretical basis contributes to expanding the understanding and development of transdiagnostic theories in promoting children and adolescents' mental health.

## Conclusion

The present systematic review aims to provide an overview of universal school-based interventions for promoting mental health in children and adolescents. The evidence gathered suggests that these interventions have a small to medium effect on a wide range of factors influencing mental health. The effectiveness of universal school-based transdiagnostic interventions is supported by the observed improvements across various mental disorders and the identification of shared mechanisms that impact different aspects of mental health.

A critical discussion within the review highlights the existing separation between the concepts of mental health promotion and mental disorders prevention. It suggests that merging the advantages of both approaches could lead to better outcomes. However, due to the lack of relevant data, a conclusive comparison between teacher-led and psychologist-led delivery of interventions could not be made. The review acknowledges that its analyses were based on desk research and may not capture the real-world challenges of conducting intervention research in school settings. Therefore, it emphasizes the need for more experimental studies to investigate and validate the suggested orientations provided by the review.

In summary, the present systematic review provides insights into universal school-based interventions for promoting mental health in children and adolescents. It underscores the positive effects observed across various mental health domains and emphasizes the potential benefits of integrating mental health promotion and mental disorders prevention approaches. While acknowledging the limitations of desk-based research, the review calls for more empirical studies to further explore and validate the suggested orientations for intervention delivery.

## References

[1] Luo Cui Z, Zou P, Wang K, Lin Z, He J, Wang J (2020). Mental health problems and associated factors in Chinese high school students in Henan province: a cross-sectional study. Int J Environ Res Public Health.

[2] Connery HS, Korte FM, McHugh RK (2020). Suicide and substance use disorder. Psychiatr Ann.

[3] Tangcharoensathien V, Witthayapipopsakul W, Panichkriangkrai W, Patcharanarumol W, Mills A. Health systems development in Thailand: a solid platform for successful implementation of universal health coverage. Lancet. 2018;391(10126):1205–1223. 10.1016/S0140-6736(18)30198-3 10.1016/S0140-6736(18)30198-3 29397200

[4] Kieling Baker-Henningham H, Belfer M, Conti G, Ertem I, Omigbodun O, Rohde LA, Srinath S, Ulkuer N, Rahman A (2011). Child and adolescent mental health worldwide: evidence for action. Lancet.

[5] Wahl MS, Adelson JL, Patak MA, Possel P, Hautzinger M (2014). Teachers or psychologists: who should facilitate depression prevention programs in schools?. Int J Environ Res Public Health.

[6] Dolotina B, Turban J (2022). Phantom networks prevent children and adolescents from obtaining the mental health care they need. Health Aff.

[7] Fleming TM, Clark T, Denny S (2014). Stability and change in the mental health of New Zealand secondary school students 2007–2012: results from the national adolescent health surveys. Aust N Z J Psychiatry.

[8] Wells J, Barlow J, Stewart-Brown S (2003). A systematic review of universal approaches to mental health promotion in schools. Health Educ.

[9] Mackenzie K, Williams C (2018). Universal, school-based interventions to promote mental and emotional well-being: what is being done in the UK and does it work? A systematic review. BMJ Open.

[10] Dalgleish T, Black M, Johnston D, Bevan A (2020). Transdiagnostic approaches to mental health problems: current status and future directions. J Consult Clin Psychol.

[11] Feiss R, Dolinger SB, Merritt M, Reiche E, Martin K, Yanes JA, Thomas CM, Pangelinan M (2019). A systematic review and meta-analysis of school-based stress, anxiety, and depression prevention programs for adolescents. J Youth Adolesc.

[12] van Loon AWG, Creemers HE, Beumer WY, Okorn A, Vogelaar S, Saab N, Miers AC, Westenberg PM, Asscher JJ (2020). Can schools reduce adolescent psychological stress? A multilevel meta-analysis of the effectiveness of school-based intervention programs. J Youth Adolesc.

[13] Werner-Seidler A, Perry Y, Calear AL, Newby JM, Christensen H (2017). School-based depression and anxiety prevention programs for young people: a systematic review and meta-analysis. Clin Psychol Rev.

[14] Loevaas MES, Sund AM, Lydersen S (2019). Does the transdiagnostic EMOTION intervention improve emotion regulation skills in children?. J Child Fam Stud.

[15] Fusar-Poli P, Salazar de Pablo G, De Micheli A, Nieman DH, Correll CU, Kessing LV, Pfennig A, Bechdolf A, Borgwardt S, Arango C, van Amelsvoort T (2020). What is good mental health? A scoping review. Eur Neuropsychopharmacol.

[16] Yang B, Chen BB, Qu Y, Zhu Y (2022). The positive role of parental attachment and communication in Chinese adolescents' health behavior and mental health during COVID-19. J Adolesc.

[17] Schaeuffele C, Schulz A, Knaevelsrud C, Renneberg B, Boettcher J (2021). CBT at the crossroads: the rise of transdiagnostic treatments. Int J Cogn Ther.

[18] Mansell W, Carey TA (2009). A century of psychology and psychotherapy: Is an understanding of control the missing link between theory, research, and practice?. Psychol Psychother.

[19] Mehrdadfar M, Ghasemzadeh S, Ghobari-Bonab B, Hasanzadeh S, Vakili S (2023). Effectiveness of unified protocols for online transdiagnostic treatment on social-emotional skills and parent-child interaction in school-aged children with cochlear implants. Int J Pediatr Otorhinolaryngol.

[20] Harvey SB, Hatch SL, Jones M (2011). Coming home: social functioning and the mental health of UK Reservists on return from deployment to Iraq or Afghanistan. Ann Epidemiol..

[21] Gatto AJ, Elliott TJ, Briganti JS, Stamper MJ, Porter ND, Brown AM, Harden SM, Cooper LD, Dunsmore JC (2022). Development and feasibility of an online brief emotion regulation training (BERT) program for emerging adults. Front Public Health.

[22] Sauer-Zavala S, Gutner CA, Farchione TJ, Boettcher HT, Bullis JR, Barlow DH (2017). Current definitions of "transdiagnostic" in treatment development: a search for consensus. Behav Ther.

[23] Glashouwer KA, Vroling MS, de Jong PJ, Lange WG, de Keijser J (2013). Low implicit self-esteem and dysfunctional automatic associations in social anxiety disorder. J Behav Ther Exp Psychiatry.

[24] Holding DH (2013). Principles of training: the commonwealth and international library: psychology division.

[25] Nolen-Hoeksema S, Wisco BE, Lyubomirsky S (2008). Rethinking rumination. Perspect Psychol Sci.

[26] Pelletier-Baldelli A, Andrews-Hanna JR, Mittal VA (2018). Resting state connectivity dynamics in individuals at risk for psychosis. J Abnorm Psychol.

[27] DeRubeis RJ, Hollon SD, Amsterdam JD (2005). Cognitive therapy vs medications in the treatment of moderate to severe depression. Arch Gen Psychiatry.

[28] Christensen AB, Dyrloev K, Hoej M, Poulsen S, Reinholt N, Arnfred SM (2023). “The depressed” and “people with anxiety” therapists’ discursive representations of patients with depression and anxiety in Danish psychiatry. Health.

[29] Durlak JA, Weissberg RP, Dymnicki AB, Taylor RD, Schellinger KB (2011). The impact of enhancing students’ social and emotional learning: a meta-analysis of school-based universal interventions. Child Dev.

[30] Conley CC, Bishop BT, Andersen BL (2016). Emotions and emotion regulation in breast cancer survivorship. Healthcare (Basel).

[31] Fenwick-Smith A, Dahlberg EE, Thompson SC (2018). Systematic review of resilience-enhancing, universal, primary school-based mental health promotion programs. BMC Psychol.

[32] Craske MG, Treanor M, Conway CC, Zbozinek T, Vervliet B (2014). Maximizing exposure therapy: an inhibitory learning approach. Behav Res Ther.

[33] Roefs A, Fried EI, Kindt M (2022). A new science of mental disorders: Using personalised, transdiagnostic, dynamical systems to understand, model, diagnose and treat psychopathology. Behav Res Ther..

[34] McEvoy PM, Nathan P, Norton PJ (2009). Efficacy of transdiagnostic treatments: a review of published outcome studies and future research directions. J Cogn Psychother..

[35] Leichsenring F, Salzer S (2014). A unified protocol for the transdiagnostic psychodynamic treatment of anxiety disorders: an evidence-based approach. Psychotherapy (Chic)..

[36] Hölzel BK, Lazar SW, Gard T, Schuman-Olivier Z, Vago DR, Ott U (2011). How does mindfulness meditation work? Proposing mechanisms of action from a conceptual and neural perspective. Perspect Psychol Sci.

[37] Tan SY (2020). Unified protocol for transdiagnostic treatment of emotional disorders (UP): empirical evidence and clinical applications from a Christian perspective. J Psychol Christianity.

[38] McLean Ruork AK, Ramaiya MK, Fruzzetti AE (2023). Feasibility and initial impact of single-session internet-delivered acceptance vs change skills for emotions for stress- and trauma-related problems: a randomized controlled trial. Behav Cognit Psychother.

[39] Dindo L, Van Liew JR, Arch JJ (2017). Acceptance and commitment therapy: a transdiagnostic behavioral intervention for mental health and medical conditions. Neurotherapeutics.

[40] Villatte JL, Vilardaga R, Villatte M, Vilardaga JCP, Atkins DC, Hayes SC (2016). Acceptance and commitment therapy modules: differential impact on treatment processes and outcomes. Behav Res Ther.

[41] Keulen Matthijssen D, Schraven J, Deković M, Bodden D (2023). The effectiveness and cost-effectiveness of acceptance and commitment therapy as a transdiagnostic intervention for transitional-age youth: study protocol of a randomized controlled trial. BMC Psychiatry.

[42] Dindo L, Recober A, Marchman JN, Turvey C, O'Hara MW (2012). One-day behavioral treatment for patients with comorbiddepression and migraine: a pilot study. Behav Res Ther..

[43] Dindo LN (2014). One-day behavioral intervention for depression and impairment in patients with comorbid depression and migraine. Headache.

[44] CASEL. What is SEL? Collaborative for Academic, Social, and Emotional Learning. 2021. https://casel.org/what-is-sel/

[45] Zins JE, Weissberg RP, Wang MC, Walberg HJ (2004). Building academic success on social and emotional learning: what does the research say?.

[46] Meuret AE, Twohig MP, Rosenfield D, Hayes SC, Craske MG (2012). Brief acceptance and commitment therapy and exposure for panic disorder: a pilot study. Cogn Behav Pract.

[47] Fairburn CG, Cooper Z, Shafran R (2003). Cognitive behaviour therapy for eating disorders: a "transdiagnostic" theory and treatment. Behav Res Ther.

[48] Balow DH, Farchione TJ, Bullis JR (2017). The unified protocol for transdiagnostic treatment of emotional disorders compared with diagnosis-specific protocols for anxiety disorders: a randomized clinical trial. JAMA Psychiatry..

[49] García-Escalera J, Chorot P, Sandín B, Ehrenreich-May J, Prieto A, Valiente RM (2019). An open trial applying the unified protocol for transdiagnostic treatment of emotional disorders in adolescents (UP-A) adapted as a school-based prevention program. Child Youth Care Forum.

[50] Hertz SP, Jensen EK, Runge E, Tarp K, Holmberg TT, Mathiasen K, Lichtenstein MB (2023). Days between sessions predict attrition in text-based internet intervention of binge eating disorder. Internet Interv.

[51] Kessler RC, Benjet C, Zainal NH, Albor Y, Alvis-Barranco L, Carrasco-Tapias N, Contreras-Ibáñez CC, Cudris-Torres L, de la Peña FR, González N, Guerrero-López JB, Gutierrez-Garcia RA, Jiménez-Pérez AL, Medina-Mora ME, Patiño P, Cuijpers P, Gildea SM, Kazdin AE, Kennedy CJ, Luedtke A, Sampson NA, Petukhova MV (2023). A precision treatment model for internet-delivered cognitive behavioral therapy for anxiety and depression among university students. JAMA Psychiat.

[52] McManus F, Shafran R, Cooper Z (2010). What does a transdiagnostic approach have to offer the treatment of anxiety disorders?. Br J Clin Psychol.

[53] Sakiris N, Berle D (2019). A systematic review and meta-analysis of the Unified Protocol as a transdiagnostic emotion regulation based intervention. Clin Psychol Rev.

[54] González-Robles A, Díaz-García A, Miguel C, García-Palacios A, Botella C (2018). Comorbidity and diagnosis distribution in transdiagnostic treatments for emotional disorders: a systematic review of randomized controlled trials. PLoS ONE.

[55] Fusar-Poli P, Correll CU, Arango C, Berk M, Patel V, Ioannidis JP (2021). Preventive psychiatry: a blueprint for improving the mental health of young people. World Psychiatry.

[56] Arango C, Díaz-Caneja CM, McGorry PD (2018). Preventive strategies for mental health. Lancet Psychiatry.

[57] World Health Organization (2005). Atlas: child and adolescent mental health resources: global concerns: implications for the future.

[58] Rowling L (2009). Strengthening, "school" in school mental health promotion. Health Educ.

[59] Cuijpers P, van Straten A, Smit F, Mihalopoulos C, Beekman A (2008). Preventing the onset of depressive disorders: a meta-analytic review of psychological interventions. Am J Psychiatry.

[60] Knapp M, Ardino V, Brimblecombe N, Evans-Lacko S, Iemmi V, King D, Snell T (2016). Youth mental health: new economic evidence. Child Young People Now.

[61] Barrett PM, Pahl KM (2006). School-based intervention: examining a universal approach to anxiety management. J Psychol Couns Sch.

[62] O’Reilly M, Svirydzenka N, Adams S, Dogra N (2018). Review of mental health promotion intervention in schools. Soc Psychiatry Psychiatr Epidemiol.

[63] Johnson C, Wade T (2021). Acceptability and effectiveness of an 8-week mindfulness program in early- and mid-adolescent school students: a randomised controlled trial. Mindfulness.

[64] Kuyken W, Weare K, Ukoumunne OC, Vicary R, Motton N, Burnett R, Cullen C, Hennelly S, Huppert F (2013). Effectiveness of the mindfulness in schools programme: non-randomised controlled feasibility study. Br J Psychiatry.

[65] Stallard P, Skryabina E, Taylor G, Phillips R, Daniels H, Anderson R, Simpson N (2014). Classroom-based cognitive behaviour therapy (FRIENDS): a cluster randomised controlled trial to prevent anxiety in children through education in schools (PACES). Lancet Psychiatry.

[66] Takahashi F, Ishizu K, Matsubara K, Ohtsuki T, Shimoda Y (2020). Acceptance and commitment therapy as a school-based group intervention for adolescents: an open-label trial. J Contextual Behav Sci.

[67] Sutan R, Nur Ezdiani M, Muhammad Aklil A (2018). Systematic review of school-based mental health intervention among primary school children. J Commun Med Health Educ.

[68] Moher D, Liberati A, Tetzlaff J, Altman DG, PRISMA Group. Preferred reporting items for systematic reviews and meta-analyses: the PRISMA statement. PLoS Med. 2009;6(7):e1000097. 10.1371/journal.pmed.1000097 10.1371/journal.pmed.1000097 PMC2707599 19621072

[69] Higgins JP, Green S (2008). Cochrane handbook for systematic reviews of interventions.

[70] Downs SH, Black N (1998). The feasibility of creating a checklist for the assessment of the methodological quality both of randomised and non-randomised studies of health care interventions. J Epidemiol Commun Health.

[71] Berry V, Axford N, Blower S, Taylor RS, Edwards RT, Tobin K, Jones C, Bywater T (2016). The effectiveness and micro-costing analysis of a universal, school-based, social–emotional learning programme in the UK: a cluster-randomised controlled trial. School Mental Health.

[72] Burckhardt R, Manicavasagar V, Batterham PJ, Hadzi-Pavlovic D, Shand F (2017). Acceptance and commitment therapy universal prevention program for adolescents: a feasibility study. Child Adolesc Psychiatry Mental Health.

[73] Cook CR, Frye M, Slemrod T, Lyon AR, Renshaw TL, Zhang Y (2015). An integrated approach to universal prevention: independent and combined effects of PBIS and SEL on youths' mental health. Sch Psychol Q.

[74] Dowling K, Simpkin AJ, Barry MM (2019). A cluster randomized-controlled trial of the mindout social and emotional learning program for disadvantaged post-primary school students. J Youth Adolesc.

[75] Dvořáková K, Kishida M, Li J, Elavsky S, Broderick PC, Agrusti MR, Greenberg MT (2017). Promoting healthy transition to college through mindfulness training with first-year college students: Pilot randomized controlled trial. J Am Coll Health.

[76] Flynn D, Joyce M, Weihrauch M, Corcoran P (2018). Innovations in practice: dialectical behaviour therapy—skills training for emotional problem solving for adolescents (DBT STEPS-A): evaluation of a pilot implementation in Irish post-primary schools. Child Adolesc Mental Health.

[77] Kishida K, Hida N, Ishikawa S (2022). Evaluating the effectiveness of a transdiagnostic universal prevention program for both internalizing and externalizing problems in children: two feasibility studies. Child Adolesc Psychiatry Mental Health.

[78] Knight MA, Haboush-Deloye A, Goldberg PM, Grob K (2019). Strategies and tools to embrace prevention with upstream programs: a novel pilot program for enhancing social and emotional protective factors in middle school students. Child Sch.

[79] Lam K, Seiden D (2020). Effects of a brief mindfulness curriculum on self-reported executive functioning and emotion regulation in Hong Kong adolescents. Mindfulness.

[80] Mendelson T, Tandon SD, O'Brennan L, Leaf PJ, Ialongo NS (2015). Brief report: moving prevention into schools: the impact of a trauma-informed school-based intervention. J Adolesc.

[81] Sawyer MG, Pfeiffer S, Spence SH, Bond L, Graetz B, Kay D, Patton G, Sheffield J (2010). School-based prevention of depression: a randomised controlled study of the beyondblue schools research initiative. J Child Psychol Psychiatry.

[82] Shum AKY, Lai ESY, Leung WG, Cheng MNS, Wong HK, So SWK, Law YW, Yip PSF (2019). A digital game and school-based intervention for students in Hong Kong: quasi-experimental design. J Med Internet Res.

[83] Skryabina E, Taylor G, Stallard P (2016). Effect of a universal anxiety prevention programme (FRIENDS) on children's academic performance: results from a randomised controlled trial. J Child Psychol Psychiatry.

[84] Torok M, Rasmussen V, Wong Q, Werner-Seidler A, O'Dea B, Toumbourou J, Calear A (2019). Examining the impact of the good behaviour game on emotional and behavioural problems in primary school children: a case for integrating well-being strategies into education. Aust J Educ.

[85] Volkaert B, Wante L, Loeys T, Boelens E, Braet C (2021). The evaluation of boost camp: a universal school-based prevention program targeting adolescent emotion regulation skills. School Mental Health Multidisciplinary Res Practice JNo Pagination Specified-No Pagination Specified.

[86] Cohen J (1988). Statistical power analysis for the behavioral sciences.

[87] Hedges LV (1981). Distribution theory for Glass's estimator of effect size and related estimators. J Educ Stat.

[88] Johnson C, Wade T (2019). Piloting a more intensive 8-week mindfulness programme in early-and mid-adolescent school students. Early Interv Psychiatry.

[89] García-Escalera J, Valiente RM, Sandín B, Ehrenreich-May J, Prieto A, Chorot P (2020). The unified protocol for transdiagnostic treatment of emotional disorders in adolescents (UP-A) adapted as a school-based anxiety and depression prevention program: An initial cluster randomized wait-list-controlled trial. Behav Ther.

[90] Bradshaw CP (2013). Preventing bullying through positive behavioral interventions and supports (PBIS): a multitiered approach to prevention and integration. Theory Pract.

[91] Ojio Y, Foo JC, Usami S (2019). Effects of a school teacher-led 45-minute educational program for mental health literacy in pre-teens. Early Interv Psychiatry.

